# The potential of biofuels from first to fourth generation

**DOI:** 10.1371/journal.pbio.3002063

**Published:** 2023-03-30

**Authors:** Philipp Cavelius, Selina Engelhart-Straub, Norbert Mehlmer, Johannes Lercher, Dania Awad, Thomas Brück

**Affiliations:** 1 Werner Siemens-Chair of Synthetic Biotechnology, TUM School of Natural Sciences, Technical University of Munich (TUM), Garching, Germany; 2 Chair of Technical Chemistry II, TUM School of Natural Sciences, Technical University of Munich (TUM), Garching, Germany

## Abstract

The steady increase in human population and a rising standard of living heighten global demand for energy. Fossil fuels account for more than three-quarters of energy production, releasing enormous amounts of carbon dioxide (CO_2_) that drive climate change effects as well as contributing to severe air pollution in many countries. Hence, drastic reduction of CO_2_ emissions, especially from fossil fuels, is essential to tackle anthropogenic climate change. To reduce CO_2_ emissions and to cope with the ever-growing demand for energy, it is essential to develop renewable energy sources, of which biofuels will form an important contribution. In this Essay, liquid biofuels from first to fourth generation are discussed in detail alongside their industrial development and policy implications, with a focus on the transport sector as a complementary solution to other environmentally friendly technologies, such as electric cars.

## Abstract 2

Biofuels have been developed to meet the increasing need for sustainable energy while reducing CO2 emissions. This Essay discusses the different types of biofuels and provides policy recommendations to accelerate the global implementation of advanced biofuels.

## Introduction

For decades, global energy demand is on the rise due to economic growth and a rapidly growing world population. Additionally, the standard of living is increasing worldwide, in most cases correlating with increased energy consumption, as energy is needed in almost every aspect of our lives, including land, water, and air transport as well as in agriculture, commercial, industrial, and domestic sectors [1]. To date, fossil fuels account for around 80% of the world’s energy demand [2], despite being a major instigator for global warming, representing roughly 89% of total greenhouse gas (GHG) emissions in 2020 [3]. Additionally, fossil fuels are predicted to deplete with the steadily increasing energy demands. As petroleum demand is constantly on the rise, estimations predict a shortage by 2070 to 2080 [4]. To that end, distinct biofuel types such as liquid and biogas should be methodologically and strategically developed as a preventive measure against predicted energy shortages, all while reducing the anthropogenic climate impact and preserving the environment.

Currently, biofuels are categorized as first to fourth generation, depending on feedstock and/or biosynthetic platform (i.e., genetic engineering). In this Essay, we present comparative advantages and disadvantages among these categories, as well as fossil sources. Furthermore, the development of biofuel technologies hinges on the socioeconomic and political landscape, which can greatly benefit from policy recommendations by respective regulatory bodies. At present, the European Union has the most stringent biofuel legislation and the most ambitious climate impact goals. Hence, we focus on EU-centered development with respect to current biofuel technology platforms at various stages of industrial deployment, the legislative framework implemented in the EU, as well as policy recommendations that would accelerate academic breakthroughs toward industrial implementation. Although, our recommendations are EU-centric, many are also applicable on a global level.

### The four generations of biofuels

One alternative to fossil fuels are biofuels, which originate from organic matter and therefore can be regrown and are termed renewable. Biofuels emit less GHGs and are in general more eco-friendly (non-toxic, sulfur-free, biodegradable) than their fossil fuel predecessors [5]. Biofuels contribute to the achievement of Sustainable Development Goals 7 (affordable and clean energy) and 13 (climate action) of the United Nations [6]. Global demand for biofuels is set to grow by 41 to 53 billion liters, or 28%, over 2021 to 2026 [7]. Typically, one can find four main types of biofuel discussed in the context of fermentation: biogas, bioethanol, biobutanol, and biodiesel. The physiochemical properties of these biofuels are compared to fossil-based fuels in Table 1.

**Table 1:** Comparison of the physiochemical properties and average EU prices (2022/2023) of biofuels.

	Biogas	Bioethanol	Biobutanol	Biodiesel	Gasoline
Number of carbon atoms	1	2	4	12–20	4–12
Density [kg L^−1^]	0.00115^[^^20^^]^	0.79^[^^21^^]^	0.81^[^^21^^]^	0.88^[^^22^^]^	0.74^[^^22^^]^
Viscosity at 20°C [mm^2^ s^−1^]	-	0.5^[^^23^^]^	3.3^[^^24^^]^	5.1^[^^23^^]^–7.5^[^^22^^]^	0.6^[^^22^^]^
Octane number	-	>100^[^^22^^]^	87^[^^23^^]^	-	92^[^^22^^]^
Cetane number	-	8^[^^22^^]^	17^[^^23^^]^	56^[^^23^^] [^^22^^]^	15–20
Lower heating value [MJ kg^−1^]	23.3^[^^23^^]^	27^[^^22^^,^^23^^]^	36^[^^23^^]^	37^[^^23^^] [^^22^^]^	43.9^[^^22^^]^
Heating value [MJ L^−1^]	0.016–0.028 ^[^^25^^]^	21.06^[^^22^^]^	29.00^[^^26^^]^	32.65^[^^22^^]^	32.48^[^^22^^]^
Flash point [°C]	-	13^[^^23^^]^	35^[^^23^^]^	160^[^^23^^]^/120	<21^[^^22^^]^
Fuel equivalence [L]	-	0.65^[^^22^^]^	-	0.91^[^^22^^]^	1^[^^22^^]^
Estimated price per heating value [Euro MJ^−1^]	0.03^[^^27^^]^	0.07^[^^28^^]^	0.06^‡^ ^[^^29^^]^	0.05^[^^30^^]^	0.05^[^^31^^]^

^‡^ As biobutanol has not achieved significant market penetration, estimated price is based on conventional n-butanol. Biobutanol prices should exceed conventional prices, given the extensive and energy demanding purification process.

Biogas formation is a fairly simple process that has been utilized for several decades. It includes four stages: hydrolysis, acidogenesis, acetogenesis, and methanogenesis. Mixed microorganisms consortia and waste streams are combined in a sealed fermentation system in the absence of oxygen. During the biogas production process, microorganisms hydrolyze waste materials into sugars, peptides and amino acids, fatty acids, and to some part into acetate and hydrogen. Afterwards, acidogenic bacteria convert those intermediate products into organic acids, mainly constituting acetic acid. In addition, they produce carbon dioxide and hydrogen. In the third step, acetogenesis, acetate is formed from hydrogen and carbon dioxide produced in the previous stage. Lastly, methanogenesis follows, creating methane from the products of acetogenesis and acidogenesis [8]. These gases can then be transformed into hydrogen and/or electricity, or can be stored as biomethane in existing geological reservoirs [9]. Since the Ukraine crisis began, the resulting lack of fossil fuel availability in the EU has led to biogas being politically pushed as a substitute to natural gas [10].

Compared to gas (biogas/hydrogen), liquid fuels offer higher energy density and simplified transport and storage. This renders them more compatible with current engine and turbine technologies [11]. Most engines and turbines are designed and built for the use of liquid fuels, which makes liquid biofuels an easy drop-in solution without the need for modifying present engine technologies or infrastructure [5,12]. These gaseous fuels pose a significant safety hazard as they ignite at lower energies and are flammable over a range of concentrations, hydrogen to higher extent, requiring high level of safety procedures [13]. The low boiling point and high octane number of bioethanol allow blending with gasoline to a certain extent. The added benefits include a more complete combustion and reduced tailpipe emissions, boosting the engine performance and reducing CO_2_ emissions. It is, however, inapt for blending with diesel. Diesel engines require hydrocarbons of higher chain length and low autoignition temperature. However, biodiesel, being of similar chemical constitution, can be blended with fossil-based diesel and hence constitutes a major energy-dense liquid biofuel. A third increasingly attractive biofuel is biobutanol, which holds high promise as it displays superior properties to bioethanol such as higher energy density (25% more energy than ethanol) and usually lower water content due to increased hydrophobicity. Biobutanol is less volatile and possesses less corrosive properties, making it easier and safer to use and store [11,14–19]. More importantly, it can be blended with both gasoline, fossil-based or biodiesel at any ratio without the need of new engine technologies and might even allow complete substitution of gasoline, while the use of ethanol is only possible as additive [11,18].

**Fig 1:**
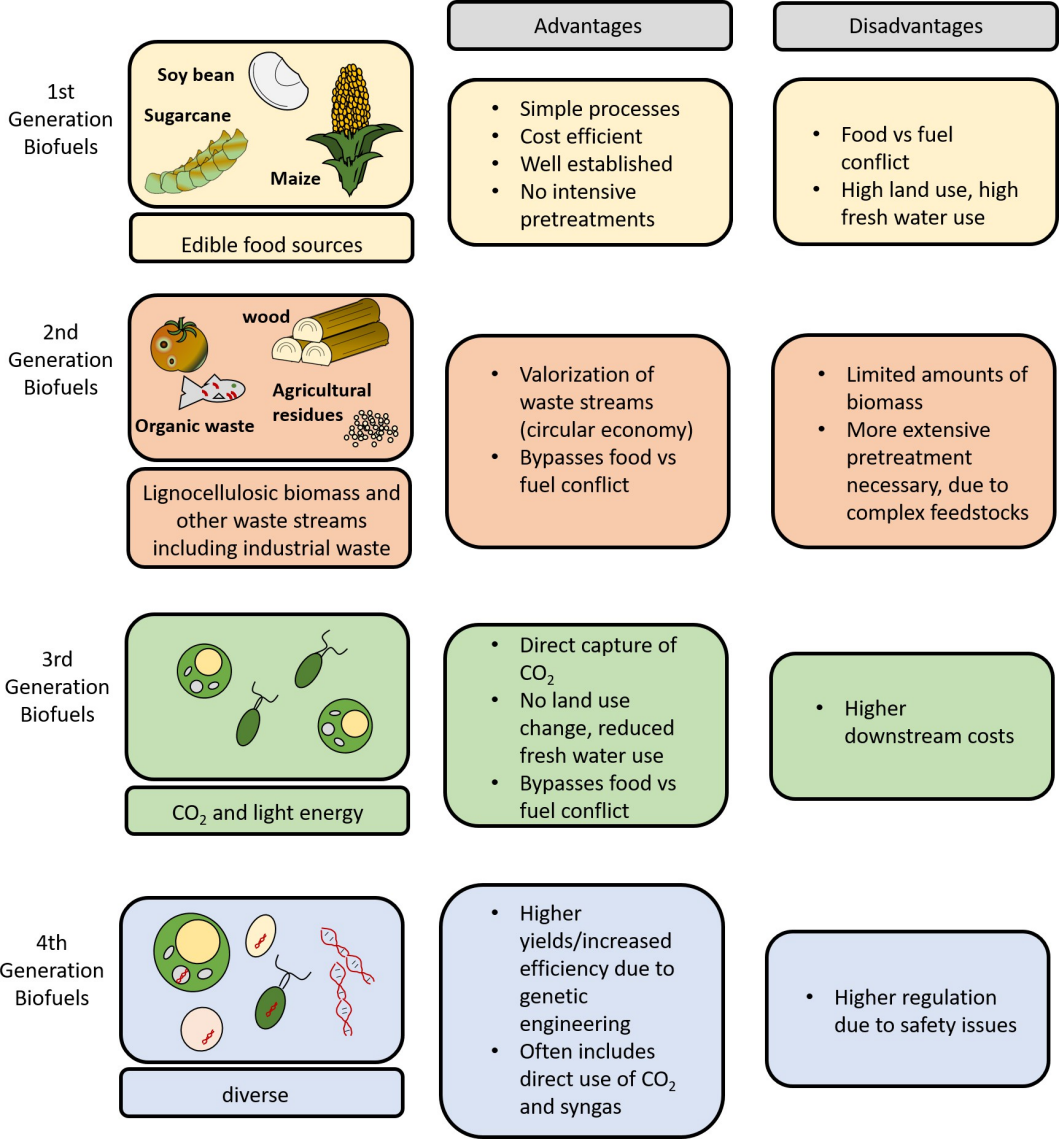
Schematic depiction of the different biofuel generations.Illustrations of possible feedstocks are depicted alongside the advantage and disadvantages associated with each generation of biofuel.

While the classification of biofuel technologies somewhat varies in the literature, products can generally be classed as first to fourth generation, depending on the type of feedstock and conversion process that was applied (Fig 1) [5].

### First-generation biofuels

Biofuels of the first generation are mainly divided into bioethanol and biodiesel. Bioethanol production of the first generation is based on microbial fermentation of edible feedstocks, rich in starch and sucrose, such as wheat, corn, and sugarcane in Europe, North America, and South America, respectively. Commercial strains include but are not limited to *Saccharomyces cerevisiae*, *S*. *stipites*, and *S*. *pombe*. Bioethanol production is not limited to first-generation biofuels; depending on the feedstock and production strain, bioethanol can also be categorized as second and third generation [32–35]. Biodiesel is mainly obtained from food-grade rapeseed, soy, or palm oil sourced from Europe, South America, and Asia, respectively. In contrast to bioethanol, it is only partially biosynthesized as its production includes chemically catalyzed steps such as transesterification of the lipids with alcohols. Enzymatic catalysis currently only exists on a lab scale [36,37]. Although biobutanol production is also possible by sugar fermentation from sugar cane, corn, wheat, and other food crops, it is limited by lower productivity and yields, product inhibition, and high costs [11,16,18,38].

During the global food demand crisis in 2007/2008, crops used for biofuel became more important to be used as food, giving rise to the “food versus fuel” debate that persists to date. Additionally, an increased demand for crops (e.g., corn) for fuel production yielded an increased market price for those foods [5]. Models predict that massive agricultural areas would be needed for fuel production and still could supply only limited amounts of fuel compared to the overall demand. It is estimated that more than two times the globally available area of arable land would needed to meet the global market demand for biodiesel when produced from rapeseed oil [39]. Furthermore, increased market values of palm oil and other biofuel cultures prompted extended deforestation of tropical rainforests for biofuel crop plantations, which releases more CO_2_ than the emission saved by those biofuels. In 2008, Fargione and colleagues estimated that it would take 319 years to repay the biofuel carbon debt resulting from clearing of tropical rainforest in Brazil and subsequent conversion to soybean plantations [40].

### Second-generation biofuels

As a result of the issues of the first generation, second-generation biofuels were developed, utilizing lignocellulosic biomass from agricultural and woodland residues as well as other waste streams (for example, from food industry like wheat bran, animal fats, or wastes of cooking and frying oil). Other non-food plants like the drought-resistant shrub or tree *Jatropha curcas*, which can also be grown in wastelands, might yet be a different promising source for second-generation biofuels [41]. Hence, second-generation biofuels circumvent the need for agricultural land use change and do not compete with food resources. However, often second-generation waste streams represent more complex feedstocks than sugarcane or palm oil, potentially containing compounds able to reduce fermentation efficiency, such as lignin. Therefore, application of additional pretreatment steps are common, increasing process time and costs [5,42,43].

For the most part, biofuels of the first and in the vast majority of the second generation are commercially produced, around 4% and 96% in 2019, respectively [44]. One example is the commercially available sunliquid from Clariant, which is a cellulosic ethanol from currently underutilized agricultural residues, such as straw. The first commercial ethanol plant in Romania started production in 2022, with plans to convert 250,000 tons of locally sourced agricultural residues to 50,000 tons of ethanol per year. After enzyme production, which hydrolyses cellulose and hemicellulose to sugar monomers, optimized microorganisms are used in fermentation to produce ethanol. These microorganisms can utilize various carbon sources like glucose and xylose, ensuring higher yields and enabling high efficiency and flexibility in waste valorization as more building blocks of waste streams can be converted to product [45]. Alongside ethanol producers, the production of second-generation biodiesel is possible from microbial lipids produced by organisms, such as *Cutaneotrichosporon oleaginosus*, a yeast capable of producing up to 90% (w/w) lipids per biomass in a fermentation process, which can be grown on residue streams (e.g., wheat bran hydrolysate medium) [46–49]. Second-generation biodiesel can also be sourced from waste oils via catalytic cracking and hydrogenation. Drawbacks of this process include incomplete conversion and coke formation, which leads to the deactivation of the catalyst. [50,51]. Biobutanol production on lignocellulose biomass and other waste streams is most commonly based on *Clostridia* fermentation, as it is one of the oldest and best-established fermentative processes for butanol production. Many *Clostridia* are natural butanol producers and possess the ability to metabolize a variety of different substrates. However, similar to its first-generation predecessor, the process is limited by low butanol titers and product inhibition [11,16,18,38]. Typically, butanol is produced via ABE fermentation, which results in solvents in ratio of 3 parts acetone, 6 parts butanol, and 1 part ethanol, and butanol refinement is not an energetically favorable solution. Other drawbacks also include cell toxicity at low concentration [52,53]. To that end, cell-free isobutanol biosynthesis using a designed artificial metabolic pathway has been developed [54]. At present, this approach remains costly for commercialization.

Various carbonaceous compounds can be transformed to syngas by gasification. Commonly, it is a gaseous waste stream from industrial processes such as steel manufacture, in which fossil fuels are burned in the process. Syngas is a mixture mainly consisting of carbon monoxide (CO), CO_2_, and hydrogen. It can be derived from biomass, including lignocellulosic compounds, coal, animal or municipal solid waste, and industrial CO-rich gases. This gas can be metabolized by strictly anaerobic, methanogenic archaea as well as by acetogenic bacterial genera such as *Acetobacterium* or *Clostridium*, often used in syntrophic fermentations. The process is mostly focused on biosynthesis of organic acids and alcohol compounds such as acetate, ethanol, and butanol [55–57]. Advantages of syngas fermentation compared to other second-generation approaches are high feedstock flexibility as well as high rates of energy and carbon capture. Complicated pretreatments of second-generation feedstocks can be replaced with gasification, using all components of the biomass, including lignin and other recalcitrant compounds [58]. LanzaTech developed a process converting feedstocks including industrial waste streams to fuel and chemicals utilizing bacteria. They estimate a total product capacity of 600,000 metric tons as well as 1,000,000 metric tons of captured carbon per year, for all their plants combined [59]. Since 2022, a demonstration plant in Japan has turned municipal solid waste to ethanol, with a production target of 20 tons of ethanol per day [60].

More than half of the biologically stored carbon is bound in marine biomass, especially macroalgae and seagrass. Detached seagrass material is seasonally washed on beaches and shore lines; due to low biological degradation and herbivore consumption, an excess of it accumulates as waste. Estimations of up to 40 million tons of dry seagrass biomass, which can be used for biofuel production, are given. Through enzymatic hydrolysis, the carbohydrate content of the seagrass can be used in a fermentation medium for microorganisms, additionally offering low nitrogen and phosphorus content, which is typically required for lipid production [61].

Despite the highly favorable ability to valorize waste streams, second-generation biofuels by themselves will not be sufficient to supply energy for the current worldwide demand. As is the case for food crops with first-generation biofuels, biomass used in these processes is available in limited amounts. Therefore, second-generation biofuels must be combined with other technologies to ensure sufficient provision of fuels. This prompted research on third-generation biofuels. However, scientific estimations predict second-generation biofuels could supply up to 30% of the world’s transportation energy [5].

### Third-generation biofuels

Third-generation biofuels are mainly derived from microalgae and cyanobacteria biomass, which can be used to naturally generate alcohols and lipids to transform into biodiesel or any other high energy fuel product. Algae exhibit 2- to 4-fold higher photosynthesis rates than terrestrial plants, resulting in faster biomass formation [62]. Algae do not require arable land or fresh water for cultivation. Many cultures can be grown using waste water, brackish or salt water, which is cost efficient and circumvents competition with agricultural activity [63,64]. Most importantly, efficient algae cultivation requires a direct CO_2_ supply, which can be derived from industrial emitters or by atmospheric carbon capture. In conventional cultivation systems, around 70% of supplied CO_2_ is used for photosynthesis and therefore biomass production [65]. Hence, algae biofuels potentially could have a negative carbon footprint as they directly bind the GHG in their biomass. One of the most prominent third-generation processes is the production of biodiesel or other energy density biofuels, such as biokerosene, using oleaginous microalgae [66,67].

**Fig 2:**
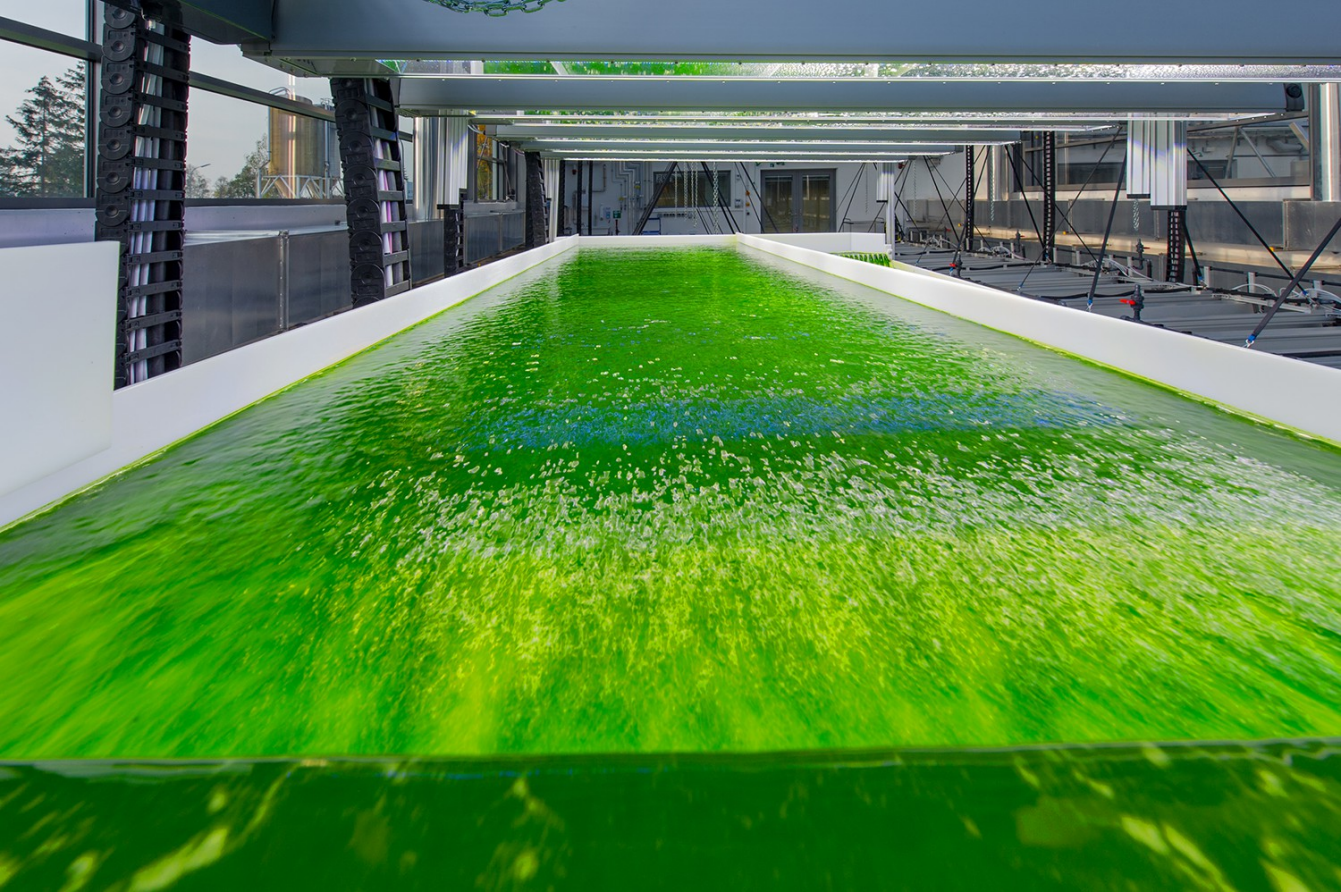
Algae cultivation at the AlgaeTec. This image showcases the open algae cultivation systems located at Technical University of Munich, Ottobrunn.

One of the most economically critical and versatile operations in algal biofuel production is algae cultivation. Algal bioreactors (Fig 2) are independent of location and climate, therefore can be operated almost irrespective of these factors. For low price, high volume products, such as biofuels, algae are commonly cultivated in open ponds. Open pond reactors are significantly cheaper in their construction and operation but have drawbacks like high loss of water through evaporation and lack of temperature control, which lowers biomass productivity. The alternative, preferred for high price, low volume products, such as cosmetic ingredients, is a closed photobioreactor, where process parameters can be precisely controlled, which often leads to higher productivity [63,68]. These bioreactors also enable a three-dimensional mode of cultivation, significantly increasing the productivity per area. In contrast to second-generation biofuels, the third-generation processes completely decouple biofuel production from the need for agricultural land. Additionally, algal-based oil production is likely greater than that in higher plants, as lipids mainly accumulate in specific parts of the plant (e.g., in rape seeds), while in algae, each cell can contain high amount of lipids, making the process more mass efficient. One bottleneck in production is harvesting, as the low size and density of the microalgal cells combined with the sensitivity of the cells to changes in pH render it challenging. [66]. Furthermore, downstream processing for algal biofuels is commonly more energy intensive than other biofuel productions [63,69]. Araújo and colleagues mapped 447 algae and cyanobacteria Spirulina production units in 2021 in the EU [70]. Most of these companies directed their biomass to the production of food, feed, and related uses; commercial application of biofuels only had a very small share. Further technological developments in upscaling and reduction of production costs are necessary for commercialization.

### Fourth-generation biofuels

The latest biofuel generation, termed fourth-generation biofuels, encompasses the use of genetic engineering to increase desired traits of organisms used in biofuel production. This applies to a variety of traits from utilizing multiple types of sugars (e.g., pentoses and hexoses), to higher lipid synthesis or increased photosynthesis and carbon fixation. For model organisms, such as *Escherichia coli* and *Saccharomyces cerevisiae*, a wide variety of tools for genetically engineering the regulation of endogenous pathways or inserting new pathways are reported. Unfortunately, for most native producers of biofuels, the genetic engineering toolbox is far more limited.

Currently, two different approaches have been adopted: engineering of pathways in native producers (optimizing growth rates, utilization of different carbon sources, directing the metabolic flux toward biofuel production and increased production titers) and reconstruction of pathways identified in natural producers in more genetically accessible model organisms. A wide variety of microorganisms can be used as heterologous hosts for the production of biofuels, including bacteria, yeast, and algae. Their metabolic versatility enables the use of various substrates to produce a wide range of biofuels. For example, butanol pathway genes from *Clostridia* were introduced into *E*. *coli*, *Pseudomonas putida*, and *Bacillus subtilis* strains [14,16,19]. While the introduction of heterologous genes is well established, a major challenge is the disruption of competing metabolic fluxes. Another obstacle for high product titers can be toxicity of large amounts of product on the cell. To enable increased accumulation of biofuels, the cellular stress response can be modified through genetic engineering, for example, with cell membrane modifications. Through the overexpression of certain membrane transporters, biofuel molecules can be secreted into the medium thereby circumventing accumulation as well as toxicity while simultaneously simplifying product recovery. In *E*. *coli*, membrane transporters have been used successfully to excrete n-alkanes, such as n-octane [71,72]. However, the overexpression of transporters is challenging as it modifies the membrane composition, creating a metabolic burden as well as potentially overloading the cellular import and export, thereby disabling the cells ability to regulate its internal environment/homeostasis [71].

Genetically modified algae can offer higher product yields and a variety of other improvements compared to wild-type algae. In order to enhance photosynthetic efficiency, the antennae systems of algae capable of absorbing a broader range of the light spectrum could be transferred to more suitable production organisms [44,73]. With respect to genetic engineering, CRIPSR/Cas9 is a frequently used tool, as it offers a simple design with efficient transfection and targeted gene disruption [74].

In fourth-generation biofuel processes that focus on genetically optimized cyanobacteria, the production of ethanol, as well as other fuel products such as butanol, isobutanol, and modified fatty acids have been realized successfully [75,76]. While 1-butanol production reached titers of 300 mg/L, bioethanol titers of up to 5.5 g/L were reported [77–79].

For the efficient optimization of native producers, systems biology can offer many insights. The availability of whole-genome sequences is essential, as this information allows for the annotation of genes to their respective function and reconstruction of the innate metabolic pathways, which can subsequently be modified. Recent advances have been made in the field of genome sequencing allowing for a more rapid and cost-efficient collection of data [19], while the gene expression patterns in different growth environments can be analyzed by transcriptomics and protein products identified by proteomics.

With genetic engineering tools, the quantity and quality of biofuels can be controlled and increased but will need political acceptance and support to be widely adopted [5]. There is a controversial debate around genetic engineering in agriculture and medicine, especially in Europe; therefore, similar concerns can be anticipated surrounding the use in biofuel production. A European-based study came to the conclusion that genetically engineered algae for biofuel production would be accepted by the majority of consumers, when the safety of the systems can be guaranteed [80]. However, with proper containment methods and carefully selected locations, such risks could be drastically minimized. Therefore, closed production systems with high security standards are expected to be built [80]. Additional biocontainment methods can be directly based on genetic changes inside the production cells such as auxotrophies or kill switches, significantly decreasing the risk of genetically modified organism (GMO) escape [44,81].

One alternative to targeted genetic engineering is random mutagenesis, which can be described as accelerated evolution. Microorganisms and products generated by this approach are not subjected to GMO regulations. Furthermore, this technique can be performed with little knowledge about the production organism and production pathway. Random mutagenesis can be achieved by a variety of methods such as UV light, chemical agents, or fast neutron irradiation. For the first time, the latter was applied on *C*. *oleaginosus*, resulting in mutants with elevated lipid titers suitable for biodiesel applications. It is noteworthy that biodiesel from prominent oleaginous yeast platforms, such as *Yarrowia lipolytica*, *C*. *oleaginosus*, *Rhodosporidium toruloides*, and *Lipomyces starkeyi*, are compliant with international biodiesel standards, including US ASTM D6751 and EU standard EN 14214 [82,83].

A new, more experimental approach to fourth-generation biofuels is the production of electrobiofuels. These are based on the approach to establish new-to-nature hybrid systems, which are able to use renewable electricity and carbon sources directly for the production of commodity chemicals and biofuels, thereby enabling the conversion of solar energy into storable liquid fuel. Such a process could combine the higher photon efficiency of modern photovoltaic systems (compared to photosynthesis) with the sustainability of biofuel production, increasing overall process effectiveness [84].

### Economics of biofuels in transportation

Apart from reducing GHG emissions and air pollution, biofuel industries can contribute to energy security on a local and national scale, as it is not reliant on local reservoirs of fossil oil. Additionally, the creation of new employment and economic growth, especially in rural locations, should positively impact the social environment as well. However, to fully exploit all the positive traits of biofuels, further research and investments are necessary, as the production of biofuels requires more processing steps compared with the conventional methods of drilling into the ground to obtain crude oil, followed by refining. Therefore, at present, biofuels commonly exceed fossil fuel production costs. Furthermore, raw materials for biofuel production do not compare to crude oil in energy density, requiring far greater amounts of biomass for the same energy output compared to fossil sources. The infrastructure required for the sector of biofuel production has to be extensively developed as well. One example is the primary energy needed to run the process, which should be obtained through sustainable operations. Candidates for that include solar and wind energy among others. Thus, by reducing the overall production cost and increasing process efficiency, biofuels could become more competitive to fossil fuels. Furthermore, by-products of biofuel production should be efficiently utilized in a circular economy, which could increase cost efficiency of such processes.

**Fig 3:**
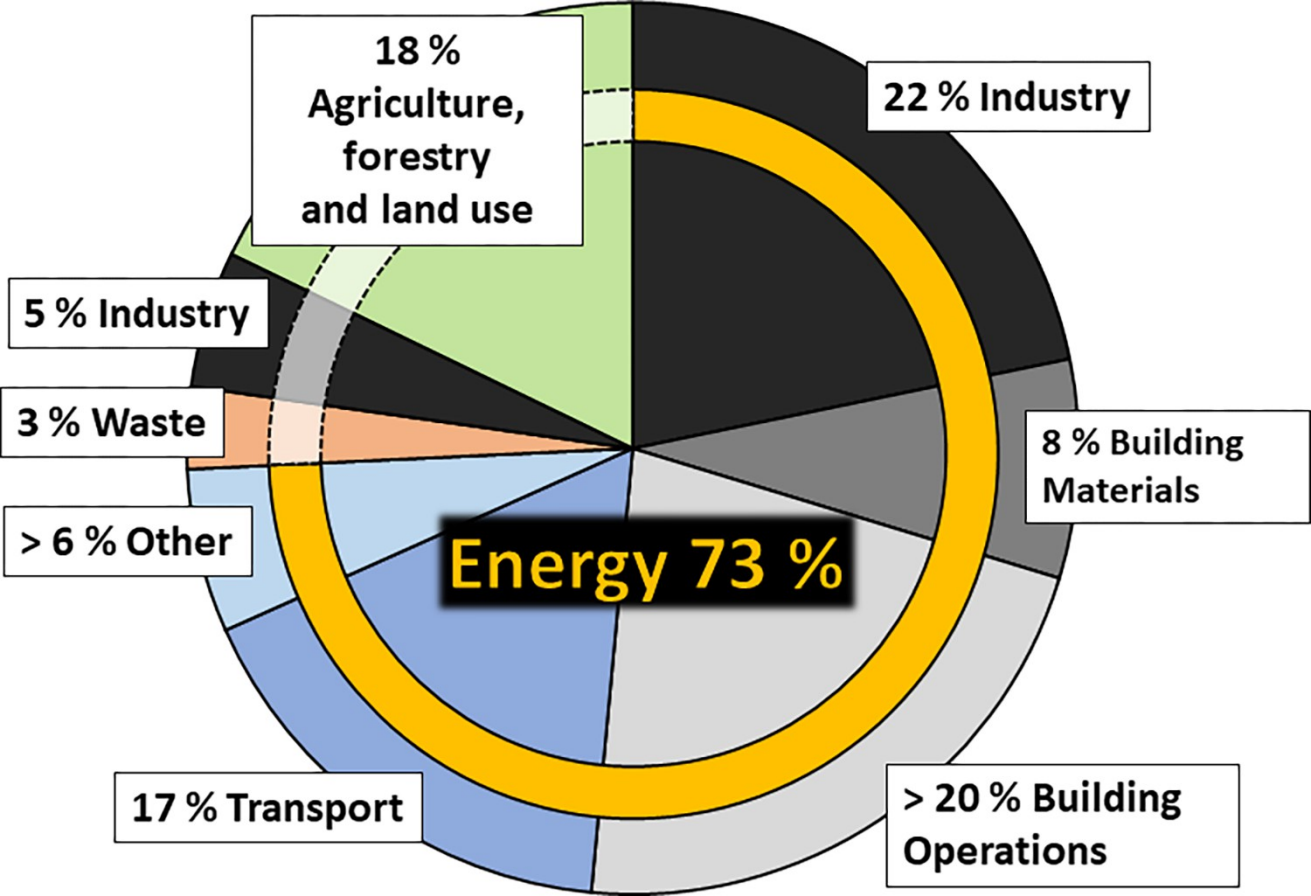
Global CO_2_ emissions divided by respective sectors. The transport sector, specifically, results in 17% of emissions. Adapted from Ritchie and colleagues (2020), Carbon Leadership Forum 2020 [85,86].

Transportation is one of the most socioeconomically sensitive sectors for the use of liquid biofuels (Fig 3). It contributes about 17% of global CO_2_ emissions [85,86], and so far, sustainable solutions are not fully developed. Due to their limitations, current technologies for biofuels are not likely to completely replace fossil fuels in their entirety but can offer new routes for waste stream valorization in a circular economy and contribute significantly to minimize our dependency on fossil fuels one step at a time. A complementary approach to this goal is electric cars, which have zero tailpipe emissions, although CO_2_ emissions are associated with the production of the car and the source of the electricity. Essential in electric vehicle batteries are metals like lithium, cobalt, nickel, and manganese. The demand for these metals is surging, while at the same time toxic waste electronics are accumulating all over the world. Traditional recycling/extraction methods require high temperatures and strong acids. This is a high energy process involving toxic chemicals. One alternative is bioleaching or biomining, which employs microbes such as *Acidithiobacillus ferrooxidans* that can bind and recover metals, bypassing the need for high temperatures and toxic chemicals [87–90]. This emerging technology offers an eco-friendly approach to recycling but still requires extensive research and development. Additionally, a new infrastructure must be put into place, supporting millions of electric cars at the same time. To that point, a combination of synthetic and biofuels in synergy with electric cars might be an optimal solution for the years to come, partially substituting fossil fuels, thereby drastically reducing CO_2_ output of transportation.

### EU policy recommendations

In order to promote the use of clean and sustainable energy at the industrial, retail, and consumer level, a cohesive framework of policies is imperative. The European Commission and European Environment Agency (EEA) have cooperated with the International Renewable Energy Agency (IRENA) and the Intergovernmental Panel on Climate Change (IPCC) in leading the efforts for clean energy transition through a number of directives and legislations since the 1990s [91–94]. These efforts manifest as a commitment by EU countries to lower GHG emissions and increase the use of renewable energy. Most notable is the Renewable Energy Directive (RED), which came into force in 2009. Through this directive, EU countries set targets for renewable energy consumption, including a subtarget mandating 10% of energy used in transport to be produced from renewables. It is noteworthy that the deployment of renewable energy has continuously grown, exceeding 22% in 2020 [92]. The legislation also mandates GHG reduction targets for fuel suppliers, requiring a reduction in GHG intensity of the fuel mix by 6% in 2020 [92]. In 2018, the commission revised the legislative proposal and the European Parliament and the EU Council proposed amendments as RED II. Therewith, the EU aims to increase the share of renewable energy to 32% and in transport to at least 14%, including a minimum share of 3.5% of advanced biofuels (second- and third-generation biofuels). The latter streamlines waste residues, such as agricultural waste (e.g., straw), and also encompasses renewable electricity in road and rail transport [95].

At present, the industrial biofuel production is dominated by first- and second-generation processes, respectively. Nevertheless, RED II and indirect land use change (ILUC) proposals have initiated the gradual shift toward second- and third-generation processes, which are associated with significant changes in feedstock supply and logistics, as well as technology deployment (e.g., market penetration of advanced biofuels). ILUC qualifies first-generation biofuels based on the unintended consequences of releasing carbon emissions as a result of land use changes [96,97]. While technical process development for third- and fourth-generation biofuels is advancing rapidly in academic and start-up settings, large-scale industrial implementation remains lagging. This indicates a profound gap in transferring technologies from a pilot scale (TRL 5) to an industrial scale (TRL 8). To that end, clear and implementable criteria remain to be addressed by legislators for industrial technology transition toward advanced biofuels with a notable climate impact. Table 2 summarizes our policy recommendations aimed at advancing biofuels implementation as well as their respective expected results and acting entity.

**Table 2:** Summary of policy recommendation and expected effects.

Priority	Action	Entity	Effect
1	Stable, long-term policies, provisions, and regulatory frameworks	Government	Incentivize and de-risk advanced technology adoption
2	Creation of structured, unified, and measurable regulatory and sustainability criteria	Government	Accelerate industrial implementation and market entry of advanced biofuel technologies via clear funding criteria
3	Create total, global carbon inventory by mapping of carbon storage and emission flux	Government	Decision guidance on feedstock trade and production plant localization
4	Pilot plants with dedicated instrumentation	Private venture/ Government	Technology validation at TRL 8 scale to accelerate market readiness
5	Sequential, stage-gated extended (4–8 years) funding periods for academic projects	Government	Concerted project development from ideation to proof of concept (TRL0–4)
6	Incentivized applied technology translation from academia to start-ups and SMEs	Private equity/ Government	Accelerate technology transfer and demonstration scale development from TRL 4–6
7	Incentivized long-term start-up financing, i.e., via Deep tech accelerator funds	Private equity/ Government	Accelerate TLR 5–8 technology optimization in industrial environment for rapid market readiness
8	Tax incentives for risk capital expenditure	Government	Accelerate technology translation and de-risk investment in new technology
9	Fair trade of feedstock and technology transfer between global north and global south	Government/ Private industry	Socioeconomic and technology-based level playingfield

First and foremost, legislators need to create stable policies and regulatory frameworks based on measurable cradle-to-cradle sustainability performance indicators. In the past, one of the greatest barriers for industry to adopt new biofuel technologies, at least in the EU, was the constantly changing regulatory and provisions framework, which ultimately led to waves of market and company consolidation for first-generation fuels such as crop-based biodiesel, corn and sugar beet-based bioethanol, and, more recently, corn-based biogas products. Therefore, it is of the utmost importance that policy makers provide clearly formulated, long-term stable policies, provisions, and regulatory frameworks to allow industrial transition to advanced biofuel technologies with clear climate impact.

With respect to sustainability, measurable criteria can be categorized as agriculture biomass, forest biomass with respect to biodiversity, and carbon stocks and emissions. Biofuel ILUC factors could be included in the biannual reports of fuel suppliers and EU countries. Accordingly, biofuel produced from palm oil and soy should carry a high ILUC factor and phasing out these feedstocks could be achieved by encouraging the diversification of feedstock. Reports estimate that 130,000 to 210,000 hectares of deforestation, which has detrimental effect on biodiversity and soil quality, could be avoided by limiting the demand of EU countries for palm oil biofuels [98]. Land requirement and fresh water use, carbon trading, and carbon offsets should also be factored in upcoming legislations. The criteria should also include GHG emissions that take the levels of methane, nitric oxides, and sulfur oxides into account in addition to levels of CO_2_. Legislation criteria should also take into consideration end-use performance, whereby industry sector, energy efficiency, and socioeconomic impact could represent qualifying measures. Risk determination and possible exceptions could be evaluated for specific industries, such as security and electricity. With respect to energy efficiency, it should be considered that distinct biofuels differ in their output. For example, ethanol yields 25% more energy than that invested in its production, while biodiesel yields 93% more [99]. To that end, performance-based renewable energy policies are needed. Finally, a reliable system that verifies compliance and reporting is eminent to putting these proposals into practice. In that respect, a mass balance system that observes the global carbon inventory and defines optimal distribution of energy profiles (first to fourth generation) and mixtures (e.g., E10 petrol/ethanol) to ensure minimal climate impact is in order. This system could integrate a range of parameters, including flexible distribution channels, demand management, storage, and price signals in real time [97,100]. Independent auditing services could further ensure compliance, which could also be extended to trading partners of the EU countries at a later stage.

As the implementation of industrial biofuel production sites are associated with immense capital investments, it is crucial to shed light on the financial aspect linked to these policies, primarily, multilevel incentives schemes, investment risk reduction, and infrastructure and logistics. On an EU level, specific funding mechanisms such as European Innovation Council (EIC) pathfinder, EIC Transition, and EIC Accelerator that aim to enable and accelerate the scaling trajectory of new technologies toward market entry already exist. While this is an initial step toward implementing new biofuel technologies, these measures do not translate into national actions and legislation on a member state level, which impedes the regional mobilization of capital, leading to a slow uptake and implementation of new technologies. Hence, a significant step toward rapid technology adoption and implementation would be the regional implementation of funding and capital mobilization as already practiced on the EU level.

An integral element in promoting advanced biofuels could be incentivizing biofuel processes that show favorable sets of sustainability parameters and end-use performance by a higher cost of CO_2_ certificates, which realistically should be in the order of 500 to 1,000 Euros/ton CO_2_. Consolidated long-term measures would also provide companies and investors with valuable tools to calculate return of investment and hence de-risk decision-making for iterative technology transition. To enable more efficient technology transfer from academia toward industrial technology deployment, additional factors need to be considered. To that end, academic projects should receive sequential, stage-gated extended funding periods of 4 to 8 years that commonly go beyond a single governmental administration period. This would allow ideas to be developed toward a proof of concept stage, where they can be translated to spin-outs or industry partners. Governments should incentivize start-up formation derived from academic units using focused funding measures, such as the EXIST funding program in Germany [101]. As technology development from proof of concept (TRL 2 to 4) in academic settings to pilot plant level often requires time periods exceeding 5 to 7 years, synergistic midterm private funding resources also have to be mobilized. To that end, technology familiarity, better understanding of time frames for solid technology development, and proper risk assessment are essential for private capital investors. In order to motivate private capital in the EU to accept development risks and extended time frames for return of investment in biofuel start-up companies, governments could implement tax write-offs for spent risk capital. This legislatively guided de-risking of capital investment into new technologies is already implemented in the United States of America and the United Kingdom, as well as in other, less compliance-driven, financial markets. Hence, the EU has to rapidly implement such legislative tax reliefs to secure innovation on the biofuels and other innovation and sustainability-driven sectors for added economic value and a vibrant job sector.

Capital is also short at the infrastructure and logistics level. Investments are required to construct dedicated pilot plants that allow industrial scale validation and optimization of new technologies, independent of any large-scale industrial partner. In that respect, multiple regionally decentralized pilot plants could provide dedicated instrumental parks that house state of the art fermentation and downstream processing equipment. In the case of gas fermentation, these parks could be associated with significant security measures and demand special regulatory approval and regular inspection. Accordingly, construction and operation by large national research organizations, such as Fraunhofer institutions in Germany, or private–public partnerships is recommended. Governmentally driven funding actions that enable access and use of these pilot plant facilities by innovators in the biofuels sector could further accelerate industrial deployment and market entry. In parallel to technology market readiness, the implementation of biofuels in industrial processes requires a secured feedstock supply.

Contrary to Nordic countries that are the forefront of advanced biofuel processes development, most industrialized countries in the EU with a high population density do not have sufficient land or biomass availability for large-scale biofuel production [100]. Hence, the location and feedstock supply require strategic positioning. Two routes for biofuels production are viable in the EU: a large production plant located in a region with abundant, long-term feedstock/biomass supply or secured trade routes; or a network of smaller, decentralized production facilities. In the latter case, a farm-integrated production facility with secured access to local residue streams can be envisioned. To optimize the economics of the production facilities, its location should be leveraged with maximal carbon credits in order to meet fuel market prices. To make an informed decision on the location and mode of production, a global carbon inventory map would be extremely beneficial. While we have a good overview of regional carbon emissions, there is little information on correlative carbon storage, which is mostly limited to terrestrial biomass. To that end, other carbon storage mechanisms should be considered, such as existing geological carbon (CO_2_) capture activities and marine biomass. Considering that 68% of the world population is projected to live in urban areas by 2050, it is sensible to consider urban waste streams, such as sewage sludge and food waste, as yet underutilized biomass feedstocks for biofuel production processes [102]. More generally, a map of the carbon flux resolved on a country-specific level would enable a more informed decision on the selection of process feedstock (biomass residues/CO_2_) and trading partners that could secure operation of large-scale production facilities for third- and fourth-generation biofuels. Currently, the major trading partners of the EU are Argentina, Brazil, USA, Indonesia, and Malaysia [97]. These trading practices do not ensure level field sustainability over the long term. To that end, future trading legislation should consider balanced trade between the global North and global South to ensure long-term beneficial socioeconomic impact on the stability and sustainability of feedstock and biofuel production.

## Conclusions

In this Essay, we laid out the reasoning for biofuel production as immediate and long-term measures to limit and eliminate energy and mobility-related GHG emissions. In that regard, biofuels will not be the only solution but an essential building block in a network with other physical (i.e., wind power, photovoltaic systems [103–105]) and chemical technologies (i.e., Sabatier process, Power to X [106,107]) that together can provide carbon neutral or even carbon negative energy and mobility solutions. In regard to transportation, biofuels should act in synergy with other technologies, such as electrified vehicles. In addition to biofuel manufacturing, similar processes could also be implemented in other applications. Here, algal and yeast oil can be transformed into building materials such as carbon fibers and cement additives. Via these routes, atmospheric CO_2_ can be absorbed from the environment and stored for very long periods of time. Such technologies could complement materials derived from fossil fuels or that generate large amounts of CO_2_ during the manufacturing process (e.g., steel, aluminum and concrete) [108].

We are convinced that, in the last decades, mankind has been generally too hesitant to adopt climate-centered technologies, which has put the world on a perilous pathway toward catastrophic climate change [109–111]. The destructive outcomes of this scenario have been documented in the scientific literature and are subject to numerous high level reports [112–117].

As time for action is already overdue, it is essential to act now by implementing the tools and technologies we have at hand at the present time. It is our opinion, that the only path to enable climate effective energy security and mobility is to deploy available technologies at a global scale right now. The global implementation of large-scale production infrastructure for sustainable (bio)technologies to kick-start production of renewable energy carriers and sustainable commodities is imperative in this timely development scenario. Once production with a base process has commenced, these processes can be iteratively refined or modulated at scale to evolve toward the next technology generation. This approach demands close, long-term academic and industry partnerships.

This fundamental transition toward sustainable bio-based technologies will require long-sighted, fact-driven legislative guidance and immense capital investments across the private and governmental sectors. However, it will be the only route to limit climate change effects and provide a livelihood for future societies.

With respect to governments, this means that neither ideology nor demagogically driven decision-making will protect any society from the effects of climate change. There are just no simple answers to complex, global problems. What is needed are global governmental alliances that make technocratically oriented long-sighted decisions, aiming for definitively set climate-centered outcomes even if the communication of the measures that have to be taken may not be popular on first sight.

Even outside the scientific communities, people are ready to accept change of the status quo in order to curb climate change effects and transition to a sustainable society. The question remains if the global political elites are ready to communicate and implement this change. Time is running out to maintain the global ecosystems as we know it.

## Abbreviations

EEA: European Environment Agency
EIC: European Innovation Council
GHG: greenhouse gas
GMO: genetically modified organism
ILUC: indirect land use change
IPCC: Intergovernmental Panel on Climate Change
IRENA: International Renewable Energy Agency
RED: Renewable Energy Directive
